# Intelligence-Augmented Rat Cyborgs in Maze Solving

**DOI:** 10.1371/journal.pone.0147754

**Published:** 2016-02-09

**Authors:** Yipeng Yu, Gang Pan, Yongyue Gong, Kedi Xu, Nenggan Zheng, Weidong Hua, Xiaoxiang Zheng, Zhaohui Wu

**Affiliations:** 1 College of Computer Science and Technology, Zhejiang University, Hangzhou, Zhejiang, China; 2 Qiushi Academy for Advanced Studies, Zhejiang University, Hangzhou, Zhejiang, China; Penn State University, UNITED STATES

## Abstract

Cyborg intelligence is an emerging kind of intelligence paradigm. It aims to deeply integrate machine intelligence with biological intelligence by connecting machines and living beings via neural interfaces, enhancing strength by combining the biological cognition capability with the machine computational capability. Cyborg intelligence is considered to be a new way to augment living beings with machine intelligence. In this paper, we build rat cyborgs to demonstrate how they can expedite the maze escape task with integration of machine intelligence. We compare the performance of maze solving by computer, by individual rats, and by computer-aided rats (i.e. rat cyborgs). They were asked to find their way from a constant entrance to a constant exit in fourteen diverse mazes. Performance of maze solving was measured by steps, coverage rates, and time spent. The experimental results with six rats and their intelligence-augmented rat cyborgs show that rat cyborgs have the best performance in escaping from mazes. These results provide a proof-of-principle demonstration for cyborg intelligence. In addition, our novel cyborg intelligent system (rat cyborg) has great potential in various applications, such as search and rescue in complex terrains.

## Introduction

Within the past two decades, bio-robots have been realized on different kinds of creatures, such as cockroaches [1], moths [2], beetles [3], and rats [4–7]. They are expected to be superior to traditional mechanical robots in mobility, perceptivity, adaptability, and energy consumption [8–11]. Among them, rat robots are becoming popular for their good maneuverability. To make a rat robot, a pair of micro electrodes are implanted into the medial forebrain bundle (MFB) of the rat’s brain, and the other two pairs are implanted into the whisker barrel fields of left and right somatosensory cortices (SI). After the rat recovers from the surgery, a wireless micro-stimulator is mounted on the back of the rat to deliver electric stimuli into the brain through the implanted electrodes. This allows a user, using a computer, to deliver stimulus pulses to any of the implanted brain sites remotely. Stimulation in MFB can excite the rat robot by increasing the level of dopamine in its brain, and stimulation in the left or right SI makes the rat robot feel as if its whiskers are touching a barrier. Before a rat robot is used for navigation, a training process is usually needed to reinforce the desired behaviors (i.e. moving ahead, turning left and turning right). In this process, the MFB stimulation acts as a reward as well as a cue to move ahead, and the left and right SI stimulation act as the cues to turn left and turn right respectively. In order to get the reward, the rat robot will learn to do the correct behaviors corresponding to the cues. The references [4, 12, 13] provide more details. After sufficient navigation training, the rat robot will move ahead in response to the Forward cue, turn left in response to the Left cue, and turn right in response to the Right cue. In this paper, the rat robot is referred to as *rat cyborg*.

One of the reasons that researchers take interest in developing rat cyborgs is that rats have outstanding spatial localization abilities. They can find a way in an environment by orienting themselves in relation to a wide variety of cues, including distal cues, typically provided by vision, audition, and olfaction, as well as proximal cues, typically provided by tactile, kinesthetic, and inertial systems [14]. Rats even have a well-developed magnetic compass sense for spatial orientation [15–17]. Rats do not build detailed geometrical representations of the environment. They rely on the learnt associations between external perception and the pose belief created from the self-motion cues. Studies of spatial orientation posit that rats use an “inner GPS” in hippocampus and entorhinal cortex to create a cognitive map of the environment [18–22]. Furthermore, RatSLAM, a navigation model which is inspired by rat’s brain, can perform simultaneous localization and mapping in real time on a mechanical robot [23–25].

On the other hand, machines (or computers) are efficient in numerical computation, information retrieval, statistical reasoning, and have almost unlimited storage. Different to rats, machines have their own methods to explore and learn about the environment in spatial navigation problems. They can capture many categories of information from the environment through various sensors, such as range sensors, visual sensors, vibration sensors, acoustic sensors, and location sensors. The information is saved and converted into a discrete and digital form. Then the processed information will be synthesized to map the environment by different paradigms [26, 27]. Eventually, machines can choose various searching algorithms in path planning, for example, flood-fill method, Dijkstra’s algorithm, A* search algorithm, rapidly exploring random tree, probabilistic roadmap. Machines run the sense process, map process, and decision process in real time and in parallel. A typical demonstration of machine’s navigation abilities is the micromouse competition, in which a small rat-like mechanical robot explores a 16×16 maze [28].

For a specific application, biological creatures and machines both have their own strengths and weaknesses. In this paper, we ask the question: can biological intelligence be augmented with the help of machine intelligence? To explore the question, a maze solving task was introduced, in which three kinds of subjects (computer, rats and rat cyborgs) were asked to find their ways from a predetermined starting position to a predetermined target position. It is a challenge to embed the machine’s maze solving capability into their navigation. In our experiments, the computer traversed the mazes based on an improved wall follower approach, six rats traversed 14 mazes one by one all by themselves, and then traversed the 14 mazes again in the same order with the assistance of the computer. Performance was measured by steps, coverage rates, and time spent, allowing for comparisons.

## Materials and Methods

### Subjects

Six rats (adult Sprague Dawley rats; 290 350 g) took part in the experiments over the course of one month. All of the six rats (named DH12, SV15, SV17, MV12, M01 and M03) had already gone through a sufficient navigation training process, which means they would turn left in response to the Left stimulus, turn right in response to the Right stimulus, and move ahead in response to the Forward stimulus. Note that in this study, Left and Right stimuli are used to instruct the rat cyborgs to turn left and right, as well as to prevent them from moving into dead roads. Forward stimulus is not used to instruct them to move ahead, but to reward them. In our experiments, the six rats and the six rat cyborgs are the same rats. A rat solving mazes by itself is called a rat, while the same rat solving mazes with the assistance of computer via its backpack stimulator is called a rat cyborg. This study was approved by the Ethics Committee of Zhejiang University (Agreement number: Zju201402-1-02-034). All of the rats used in these experiments were well cared for by the animal keepers. All of the experiments were performed in accordance with the guidelines issued by the Ethics Committee of Zhejiang University, and they complied with the China Ministry of Health Guide for the Care and Use of Laboratory Animals.

### Apparatus

**Fig 1:**
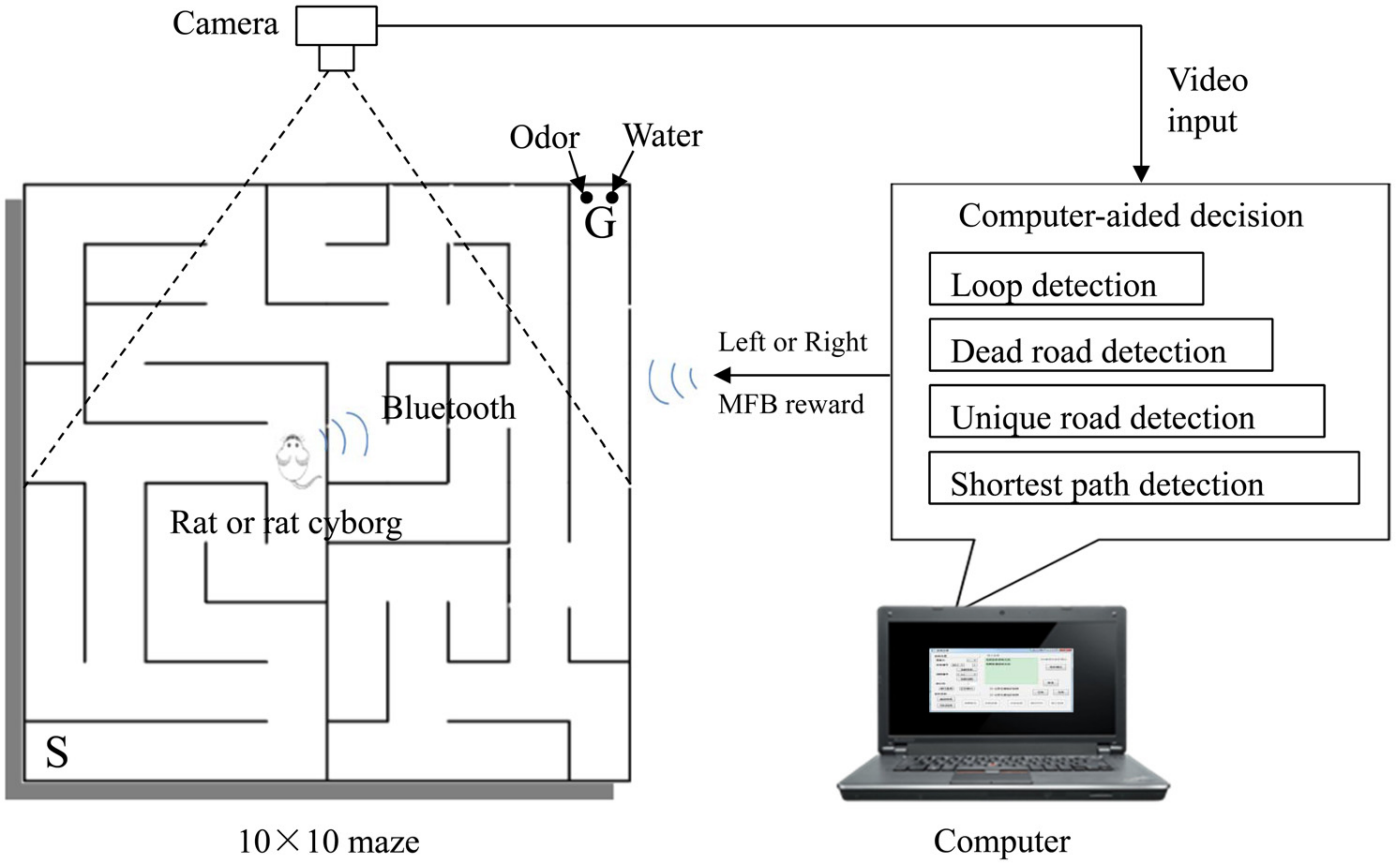
Experimental system for maze solving.

The experimental system for maze solving is shown in Fig 1. The maze is made of wood, and comprises 10×10 unit squares (15 cm×15 cm per unit square). Walls of this maze are also 15 cm high and the outside walls enclose the entire maze. The maze is covered with a piece of perspex on the top, which is used to prevent rats from climbing and escaping. The starting position S is in the bottom-left cell of the maze, and target position G is in the top-right cell of the maze. A notable feature of this maze is that the four walls of each cell can be inserted or removed. Therefore we can change the layout of each maze, and set various paths from S to G. In our experiments, we designed 14 mazes (maze 1 to maze 14, see S1 Fig) with different levels of complexity. Furthermore, there were a box of peanut butter and a dish of water in the target cell. The peanut butter was used as an odor source, and the water was used as a reward.

The whole experimental process was captured by a web camera. The camera we used could capture video clips at a rate of 15 frames per second, with a resolution of 640 by 480 pixels. The computer acquired the explored maze information and motion states of the rats in real time with the assistance of the camera. The computer-aided maze solving system running on the computer was implemented in C++ with a user-friendly interface. Left, Right and Forward stimuli were sent to the backpack of a rat via Bluetooth. Left and Right stimuli were sent to guide the rats only in the maze solving procedure by rat cyborgs. Forward stimulus acted as another reward (like water) after the rats reached the target cell in maze solving procedures by rats and by rat cyborgs.

### Procedures

#### Maze Solving Training

In this procedure, in order to get the rewards in the target cell, rats had to learn to find a path from the constant starting position to the constant target position. We used double rewards to reinforce rat’s maze solving behavior: water reward and electric reward of MFB stimulation. Water is a natural reward, which is indispensable to rats. MFB reward is a train of short-duration pulses, which can excite the rats. A combination of water reward and MFB reward can accelerate this maze solving training procedure [29, 30]. In addition, olfactory cues can be an aid for the rats in solving spatial problems [31–34]. In our experiments, a box of peanut butter was placed in the target cell (see Fig 1), which was used to help rats locate the target cell, and activate their odor tracking abilities.

The detailed training procedure is as follows. After water deprivation for 24 hours, six rats, which had been well trained in the navigation training, proceeded to the maze solving training, which consisted of 3 to 5 random mazes per day in consecutive days. The training time in each day was 8-12 a.m. and 2-6 p.m. In each maze, at first the rat was placed in the constant starting cell. It had to learn to find a path to the target cell by itself. After the rat arrived at the target cell and drank a drop of water (0.15 ml) in a dish, five consecutive MFB rewards were sent. Each rat ran each maze only one time, and each maze had a different layout. Once all of the six rats finished a maze, they proceeded to training in the next maze. Each rat was offered only 6 ml water after one day’s training. This amount of water was to meet the basic need, but not enough to completely satisfy a rat. The rat still wanted to drink water on the next day. The entire training lasted 7 days. After this procedure, all of the six rats had a strong desire to run in the mazes to search for the water and MFB rewards.

#### Maze Solving by Computer

**Fig 2:**
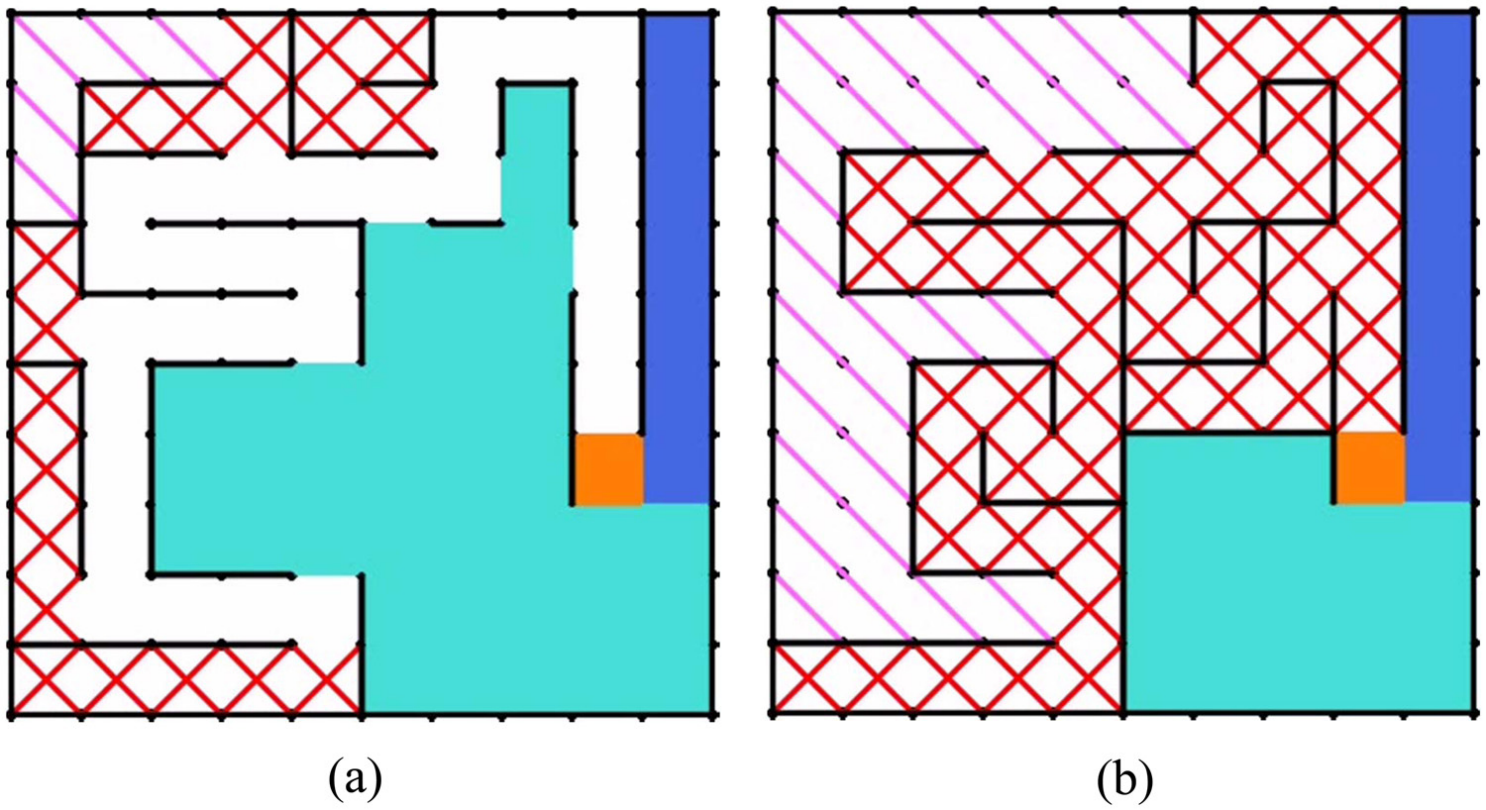
Maze solving by our algorithm. The yellow cell is the current position of the explorer. Blue cells indicate a unique road to the target. Cyan cells are cells which have not been explored, and walls of these cells now are unknown to the explorer. Pink slash (“∖”) denotes the non-visited dead road, red cross (“×”) denotes the visited dead road. (a) Left-hand. (b)Right-hand.

Similar to rats, the computer had to find a path from the start to the target with no knowledge of the mazes. There are a number of different maze solving algorithms, such as random mouse, wall follower, Pledge, and Tremaux’s algorithm [35, 36]. In this study, on the basis of dead road detection and unique road detection, two wall follower rules (left-hand and right-hand) were adopted to traverse the 14 mazes. Dead roads refer to roads that can not lead to the target. There are two kinds of dead roads: visited dead road and non-visited dead road. The dead road detection algorithm is listed in **Algorithm A in** S1 File. A unique road refers to the only way to the target. The detailed unique road detection algorithm is listed in **Algorithm B in** S1 File. The complete maze solving algorithm is listed in Algorithm 1. For left-hand rule, the traversal sequence is clockwise (left→front→right→back), and for right-hand rule, the traversal sequence is anticlockwise (right→front→left→back). Fig 2 shows two maze solving processes by our algorithm. Steps and coverage rates of maze solving in the 14 mazes were recorded. We took the average steps and average coverage rates of the left-hand rule and right-hand rule as the performance measures. Two demo videos of maze solving by our algorithm are presented (**Videos A and B in** S2 File).

Algorithm 1: Maze solving by our algorithm (left-hand).

1 **while** *the current cell C is not the target cell* **do**

2  **if** there is a unique cell U which is accessible in the four adjacent cells **then**

3   move to U;

4  **end**

5  **else if** *the left cell of C is accessible and not a dead cell* **then**

6   move to the left cell;

7  **end**

8  **else if** *the front cell of C is accessible and not a dead cell* **then**

9   move to the front cell;

10  **end**

11  **else if** *the right cell of C is accessible and not a dead cell* **then**

12   move to the right cell;

13  **end**

14  **else if** *the back cell of C is accessible and not a dead cell* **then**

15   move to the back cell;

16  **end**

17 **end**

**Fig 3:**
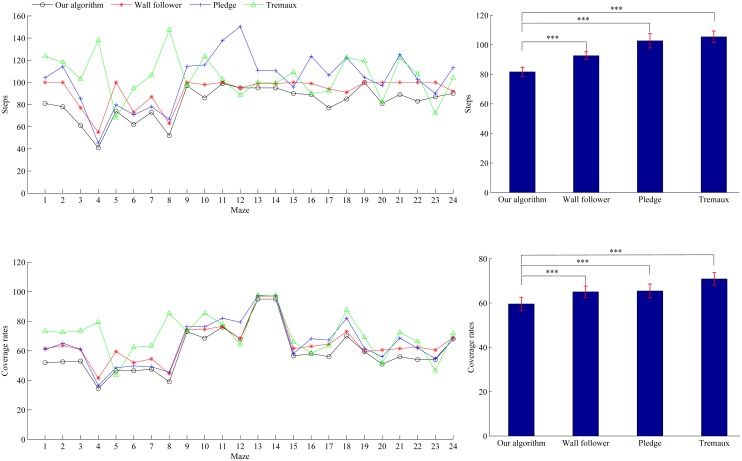
Steps and coverage rates of maze solving by our algorithm, wall follower, Pledge and Tremaux’s algorithm. Results are presented as mean±s.e.m. on the right. *p<0.05, **p<0.01, ***p<0.001.

To ensure that our algorithm can reflect the computer’s maze solving capacity, we compare its performance in 24 mazes (maze 1 to maze 24, see S1 Fig) with that of three classical maze solving algorithms (i.e. wall follower, Pledge and Tremaux’s algorithm). The experimental results are presented in Fig 3. We can see from the left column that both the steps and coverage rates of our algorithm in most mazes are less than those of the other three algorithms. From the right column, the average steps and average coverage rates of our algorithm are less than those of the other three algorithms. The statistical analysis (two-tailed paired t-test) also shows that there are significant differences of the steps and coverage rates between our algorithm and the other three algorithms (*p*<0.001). The results indicate that our algorithm outperforms the other three algorithms in maze solving. Thus we take the performance of our algorithm as computer’s performance in maze solving, and compare it with that of rats and rat cyborgs.

#### Maze Solving by Rats

After the maze solving training, the entire maze was washed and dried, and then the six rats proceeded to this formal maze solving procedure. In this procedure, the rats solved 14 mazes one by one using their own spatial learning abilities. Details of this procedure are similar to the maze solving training procedure. The experimental time in each day was 8-12 a.m. and 2-6 p.m. Each rat ran each maze only one time. In each maze, at first the rat was placed in the constant starting cell. After the rat arrived at the target cell and drank a drop of water (0.15 ml) in a dish, five consecutive MFB rewards were sent. Once all of the six rats finished a maze, they proceeded to solve the next maze. Each rat was offered 6 ml water at the end of one day’s experiment. Steps, coverage rates and time spent of maze solving by the rats in the 14 mazes were analysed. Additionally, we observed that the strategy rats took to solve the mazes was not easily understood (in other words, it was not perfect). They revisited the visited cells, explored the dead roads which could be easily detected by the computer, and even returned to the starting cell when they were confused. This offered an opportunity to the computer to help rats in maze solving. A demo video of this procedure is shown in **Video C in** S2 File.

#### Maze Solving by Rat Cyborgs

**Fig 4:**
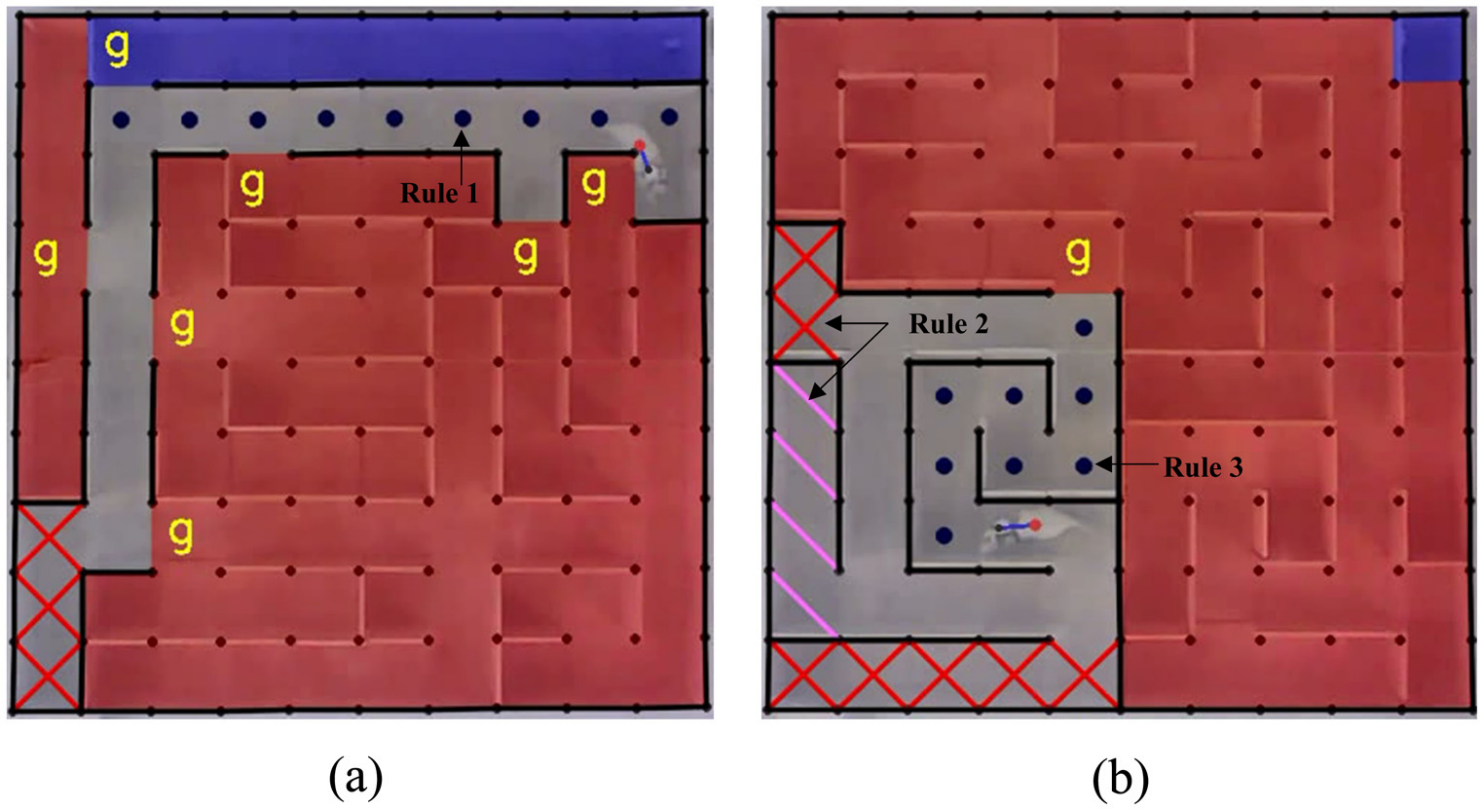
Maze solving by rat cyborgs. The red point is the body position, the black point is the head position, and the blue line is the heading direction of the rat cyborg. Our rat cyborg tracking method (see Algorithm 2) is implemented by OpenCV. Blue cells indicate a unique road to the target. Red cells are cells which have not been explored, and walls of these cells now are unknown to the rat cyborg. The pink slash (“∖”) denotes the non-visited dead road, the red cross (“×”) denotes the visited dead road, and the yellow “g” denotes entrances to the unexplored area. Consecutive blue points indicate the shortest path to the current destination. The shortest path is detected by A* algorithm [37].

A rat cyborg is a computer-aided rat, which is a combination of a rat and machines. After the procedure of maze solving by rats, the entire maze was washed and dried, and then the same six rats traversed the 14 mazes in the same order with the computer’s help. Details of this procedure are similar to the procedure of maze solving by rats, except that the rats have the help of the computer to solve the mazes. Fig 4 shows two maze solving processes by rat cyborgs. With the motivation to help rats solve the mazes, the computer tracked the rats, analyzed the explored maze information, and decided when and how to intervene. The computer aided the rats under three rules: (1) if there was a path to the unique road, the computer would find the shortest path, then Left and Right commands would be sent to navigate the rat to the unique road; (2) if the rat was going to enter a dead cell, Left or Right commands would be sent to prevent such a move; (3) if the rat was in a loop (detected by **Algorithm C in** S1 File), the computer would find the shortest path to the current destination, then Left and Right commands would be sent to navigate the rat to follow the path. In this experiment, the computer automatically analyzed the dead roads, unique roads, loop roads and shortest roads in real time, and generated the guiding information to overlay on the live video, which is playing on the screen of the computer. At the same time, an expert was watching the guiding information shown on the screen and sent the Left and Right commands to direct the rat under the three rules. The sent Left and Right commands were also shown simultaneously in the video. Please note that the expert was not directly involved in the maze solving, his/her operations to send the Left and Right commands under the guidance of the computer is just a simple replacement of the computer’s execution (action), not an augmentation or a diminution of the intelligence, since all the path detection are performed by the computer. Apart from the above three rules, rats solved the mazes of their own free will. We did not treat rats as machines which were totally controlled by the computer. Steps, coverage rates and time spent of maze solving by the rat cyborgs in the 14 mazes were analysed. A demo video of this procedure is shown in **Video D in** S2 File.

Algorithm 2: Rat cyborg tracking algorithm.

1 Save a background image to *I*_*a*_;

2 **while** *the rat cyborg has not reached the target cell G* **do**

3  pick an image *I*_*b*_ from the camera;

4  use *cvAbsDiff* to subtract *I*_*a*_ from *I*_*b*_, write the difference to *I*_*c*_;

5  use *cvThreshold* to get a binary image *I*_*d*_ from *I*_*c*_;

6  use *cvFindContours* to find all of the contours *C*_*o*_ in *I*_*d*_;

7  search the largest contour *C*_*max*_ in *C*_*o*_;

8  calculate the centroid of pixels in *C*_*max*_, write it to the body position *R*_*b*_;

9  use *cvGoodFeaturesToTrack* to detect corners in *C*_*max*_ find a 40×40 square *S*_*o*_ in *C*_*max*_;

10  find a 40×40 square *S*_*o*_ in *C*_*max*_, which has the most corners;

11  calculate the centroid of corners in *S*_*o*_, write it to the head position *R*_*h*_;

12 **end**

## Results

In this section, the experimental results of maze solving by computer, by rats and by rat cyborgs are presented. We first compare steps and coverage rates of maze solving among them. Then we compare time spent of maze solving between rats and rat cyborgs. Finally, correlation between the steps and correlation between the coverage rates are analyzed.

### Steps

**Fig 5:**
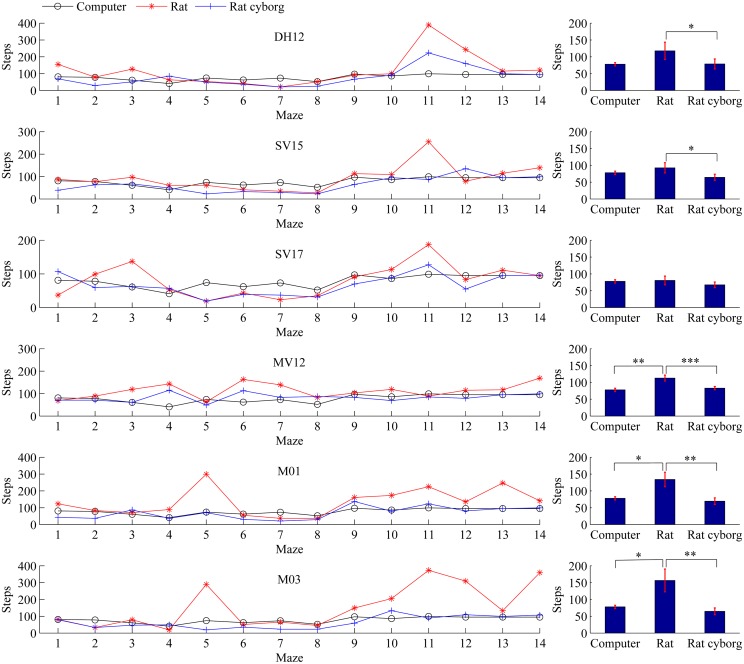
Steps of maze solving by computer, rats and rat cyborgs. The steps of maze solving by DH12, SV15, SV17, MV12, M01 and M03 are shown from the top to the bottom, respectively. Data are presented as mean±s.e.m. on the right. *p<0.05, **p<0.01, ***p<0.001.

Steps are the sum of times visiting each cell. A cell can be visited multiple times. Steps of maze solving by the six rats/rat cyborgs are presented with those by the computer in Fig 5. We can see from the left column that the steps of rat cyborgs in most mazes are less than those of rats, and may be equivalent to those of the computer. From the right column, the average steps of each rat cyborg are less than that of each corresponding rat: DH12 (from 117.71±25.70 to 78.64±14.94), SV15 (from 92.57±15.28 to 64.43±9.12), SV17 (from 80.43±12.92 to 67.36±8.41), MV12 (from 112.86±8.74 to 82.86±4.96), M01 (from 134.00±21.53 to 69.29±9.88), and M03 (from 156.43±33.92 to 64.57±10.06); and the average steps of the computer (77.79±4.90) are also less than that of each rat. The two-tailed paired t-test shows that between computer and rats, there are three rats (i.e. MV12, M01 and M03) that performed worse than the computer; between computer and rat cyborgs, there are no statistically significant differences; and between rats and rat cyborgs, there are five rats (i.e. DH12, SV15, MV12, M01 and M03) that performed better with the assistance of the computer. These results suggest that in maze solving, based on the steps, the performance of rat cyborgs is better than that of individual rats, and comparable to that of individual computer.

### Coverage Rates

**Fig 6:**
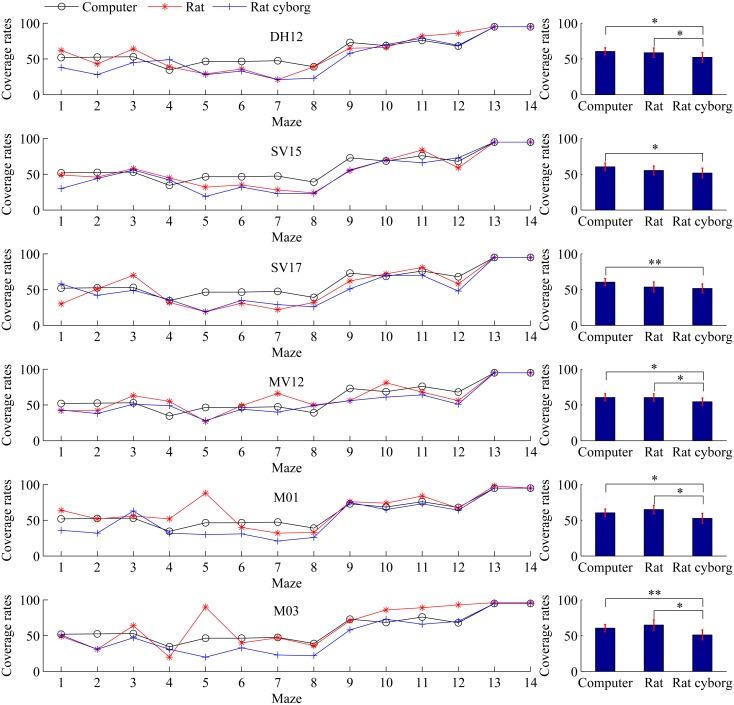
Coverage rates of maze solving by computer, rats and rat cyborgs. The coverage rates of maze solving by DH12, SV15, SV17, MV12, M01 and M03 are shown from the top to the bottom, respectively. Data are presented as mean±s.e.m. on the right. *p<0.05, **p<0.01, ***p<0.001.

Coverage rate is the number of visited cells. Every visited cell is counted only one time, so the maximum value of the coverage rate is 100. The larger the coverage rate is, the less efficient the maze solving is. It can evaluate the performance of the explorer in maze solving from another point of view. Coverage rates of maze solving by the six rats/rat cyborgs are presented with those by the computer in Fig 6. We can see from the left column that the coverage rates of rat cyborgs in most mazes are less than those of the computer and those of the rats. From the right column, the average coverage rates of each rat cyborg are less than that of each corresponding rat: DH12 (from 58.71±6.59 to 52.21±6.89), SV15 (from 55.36±6.26 to 51.71±6.84), SV17 (from 53.57±7.06 to 51.64±6.35), MV12 (from 60.36±5.25 to 54.57±5.20), M01 (from 65.07±5.86 to 52.71±6.92), and M03 (from 64.86±7.27 to 51.07±6.92); and the average coverage rates of each rat cyborg are also less than that of the computer (60.50±5.13). The two-tailed paired t-test shows that between computer and rats, there are no statistically significant differences; between computer and rat cyborgs, all of the six rat cyborgs performed better than the computer; and between rats and rat cyborgs, there are four rats (i.e. DH12, MV12, M01 and M03) that performed better with the assistance of the computer. These results suggest that in maze solving, based on the coverage rates, the performance of rat cyborgs is better than that of individual computer and that of individual rats.

### Time Spent

**Fig 7:**
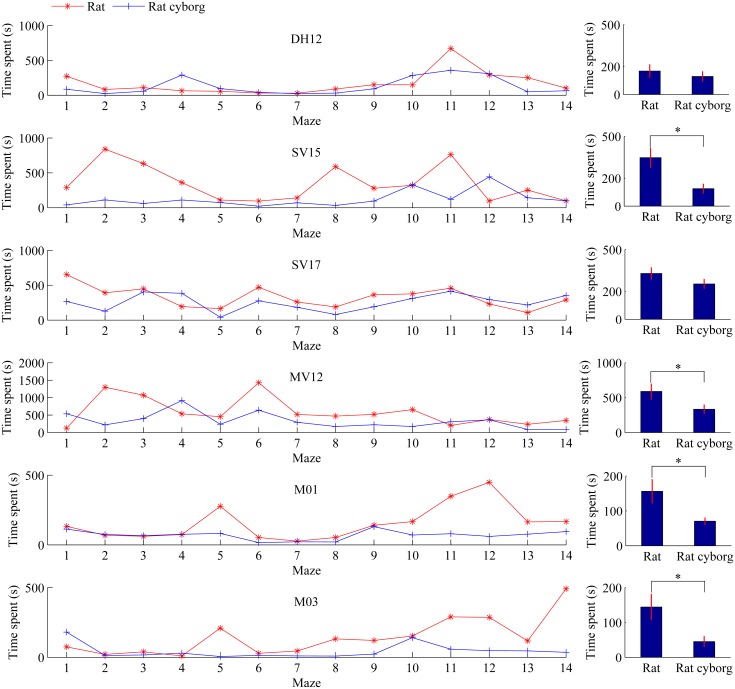
Time spent of maze solving by rats and rat cyborgs. The time spent of maze solving by DH12, SV15, SV17, MV12, M01 and M03 are shown from the top to the bottom, respectively. Data are presented as mean±s.e.m. on the right. *p<0.05, **p<0.01, ***p<0.001.

Time spent measures the period between a rat/rat cyborg starts to move at S and the rat/rat cyborg reaches G. Besides steps and coverage rates, we further compare the time spent of the rats in the 14 mazes with those of the rat cyborgs. Time spent of maze solving by the six rats/rat cyborgs are presented in Fig 7. As we can see from the left column that the time spent of rat cyborgs in most mazes are less than those of rats. From the right column, the average time spent of each rat cyborg is less than that of each corresponding rat: DH12 (from 168.07±44.95 to 129.36±32.74), SV15 (from 347.43±68.68 to 125.00±31.48), SV17 (from 328.50±40.19 to 253.43±31.69), MV12 (from 588.43±106.85 to 333.07±61.58), M01 (from 156.14±33.13 to 70.50±8.82), and M03 (from 144.36±36.35 to 45.14±13.96). The two-tailed paired t-test shows that there are four rats (i.e. SV15, MV12, M01 and M03) that performed better with the assistance of the computer. These results suggest that in maze solving, based on the time spent, the performance of rat cyborgs is better than that of individual rats.

### Correlation

**Fig 8:**
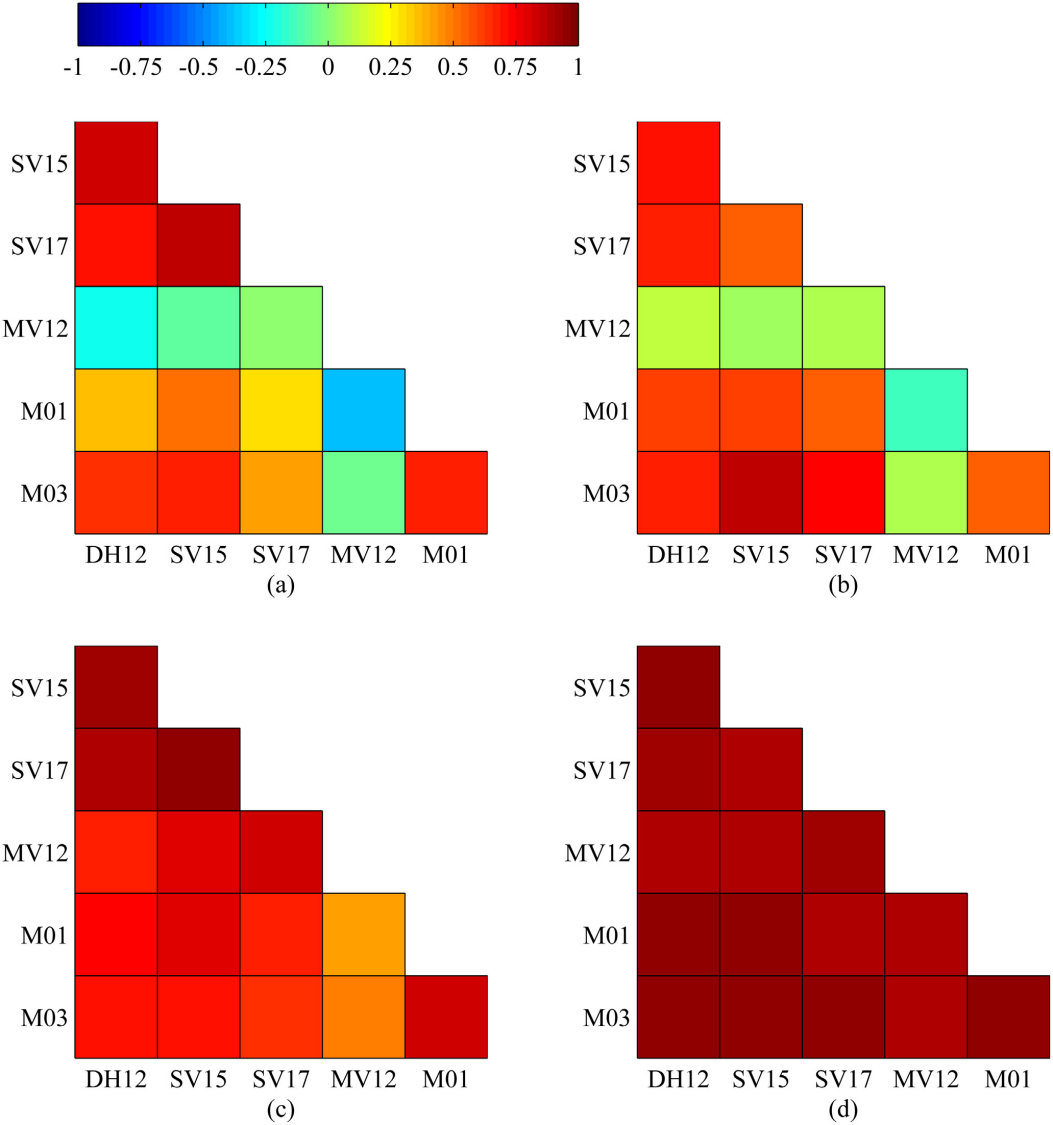
The Pearson’s correlation coefficients of steps and coverage rates between each other within the six rats/rat cyborgs. (a) Correlation coefficients of steps within the six rats. (b) Correlation coefficients of steps within the six rat cyborgs. (c) Correlation coefficients of coverage rates within the six rats. (d) Correlation coefficients of coverage rates within the six rat cyborgs.

The 14 mazes have different levels of complexity from the human perspective, and the more complicated mazes should consume more steps to solve. Maze 11 was supposed to be the most complicated maze among the 14 mazes, and it took the rats exceptionally many more steps (see Fig 5). Firstly, we analyze the Pearson’s correlation coefficients of steps and coverage rates between each other within the six rats/rat cyborgs (see Fig 8). In terms of steps, except MV12, the other five rats/rat cyborgs have a positive correlation between each other in maze solving. In terms of coverage rates, all of the six rats/rat cyborgs have a positive correlation between each other in maze solving. These results indicate that a maze that is difficult for a rat/rat cyborg should be difficult for other rats/rat cyborgs as well. Secondly, we further analyze the Pearson’s correlation coefficients of steps and coverage rates between computer and rats, computer and rat cyborgs, and rats and the corresponding rat cyborgs (see Table 1). In terms of steps, all of the correlation coefficients are strong positive, except those between MV12 and the computer. In terms of coverage rates, all of the correlation coefficients are strong positive. These results indicate that a maze which is difficult for a rat should be difficult for the corresponding rat cyborg and computer as well, and vice versa.

**Table 1:** The Pearson’s correlation coefficients of steps and coverage rates between computer and rats, computer and rat cyborgs, and rats and rat cyborgs.

	DH12		SV15		SV17		MV12		M01		M03	
	s	c	s	c	s	c	s	c	s	c	s	c
Computer vs Rats	0.5610	0.8822	0.6524	0.9141	0.4984	0.8951	−0.1137	0.7724	0.5784	0.7966	0.6979	0.7970
Computer vs Rat cyborgs	0.5696	0.9018	0.6653	0.9022	0.6034	0.9059	-0.2160	0.8667	0.7198	0.9399	0.6548	0.9376
Rats vs Rat cyborgs	0.9254	0.9224	0.5469	0.9450	0.7024	0.9005	0.7004	0.9123	0.6302	0.7881	0.5639	0.7350

s is an abbreviation for steps, c is an abbreviation for coverage rates.

## Discussion

The experimental results show that, in terms of steps, coverage rates and time spent, rat cyborgs performed better than rats in maze solving. However, in terms of steps, performance of rat cyborgs did not show remarkable advantages over that of computer. Actually, rats did not strive to reach the destination in the least number of steps. They sometimes would revisit visited cells again and again, and wander between adjacent cells. Nonetheless, all of the six rat cyborgs outperformed the computer in terms of the coverage rates. Moreover, the rat cyborgs are agile in different types of terrain (see S1 Video), and have the potential to solve unanticipated problems relying on instinct [38].

Because the six rats and the six rat cyborgs were the same rats, the possible interference should be carefully prevented. In our experiments, we took four measures to avoid interference: (1) for a rat and its rat cyborg, the layout of each maze was the same, while the physical walls of each maze cell were changed; (2) a maze solved by a rat would be solved by its rat cyborg at least 4 days later, not in the following day; (3) in the interval, the rat would be asked to carry out experiments with other mazes to further weaken its memory of the previous maze; (4) between the procedure of maze solving by rats and the procedure of maze solving by rat cyborgs, the entire maze was washed and dried to remove the possible odor interference. In order to verify that the performance enhancement of the rat cyborgs in maze solving was not attributed to what the rats had experienced, we conducted two supplemental experiments.

**Fig 9:**
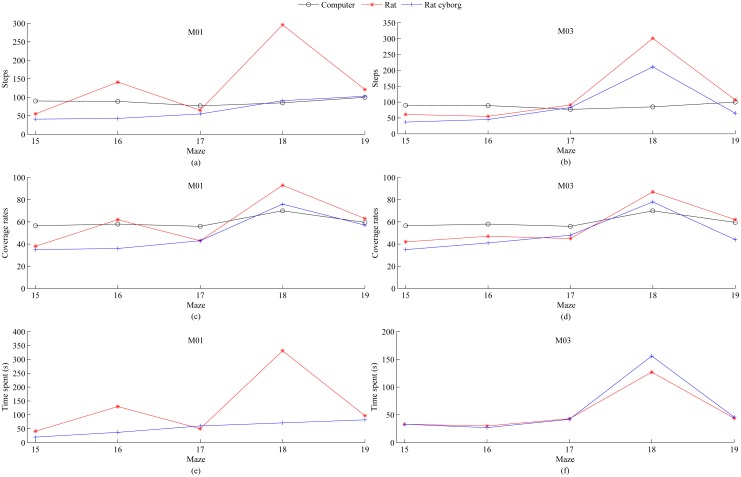
Steps, coverage rates and time spent of maze solving by computer, M01 and M03 in maze 15 to maze 19. (a) Steps of maze solving by computer and M01. (b) Steps of maze solving by computer and M03. (c) Coverage rates of maze solving by computer and M01. (d) Coverage rates of maze solving by computer and M03. (e) Time spent of maze solving by computer and M01. (f) Time spent of maze solving by computer and M03.

In supplemental experiment 1, two rats (i.e. M01 and M03) first traversed 5 mazes (maze 15 to maze 19, see S1 Fig) one by one with the assistance of the computer, and then traversed the 5 mazes again in the same order all by themselves. If a strong memory of the mazes has been gained by the rat cyborgs, it will then benefit the performance of the rats. The experimental results are shown in Fig 9. As we can see, the steps of each rat cyborg are less than that of the corresponding rat in each maze. The coverage rates of each rat cyborg are less than that of the corresponding rat except in maze 17 of M03, the coverage rates of each rat cyborg are less than that of the computer except in maze 18, and the average coverage rates of each rat cyborg (M01: 49.40±7.72, M03: 49.20±7.51) are less than that of the corresponding rat(M01: 59.80±9.68, M03: 56.60±8.35) and the computer (60.00±2.57). Besides, M01 spent less time in maze solving with the assistance of the computer except in maze 17. These results show that rat cyborgs have a better performance than rats. This is consistent with the conclusion of the previous experiments, and demonstrates that the performance enhancement of the rat cyborgs in maze solving should not be attributed to what the rats had experienced.

**Fig 10:**
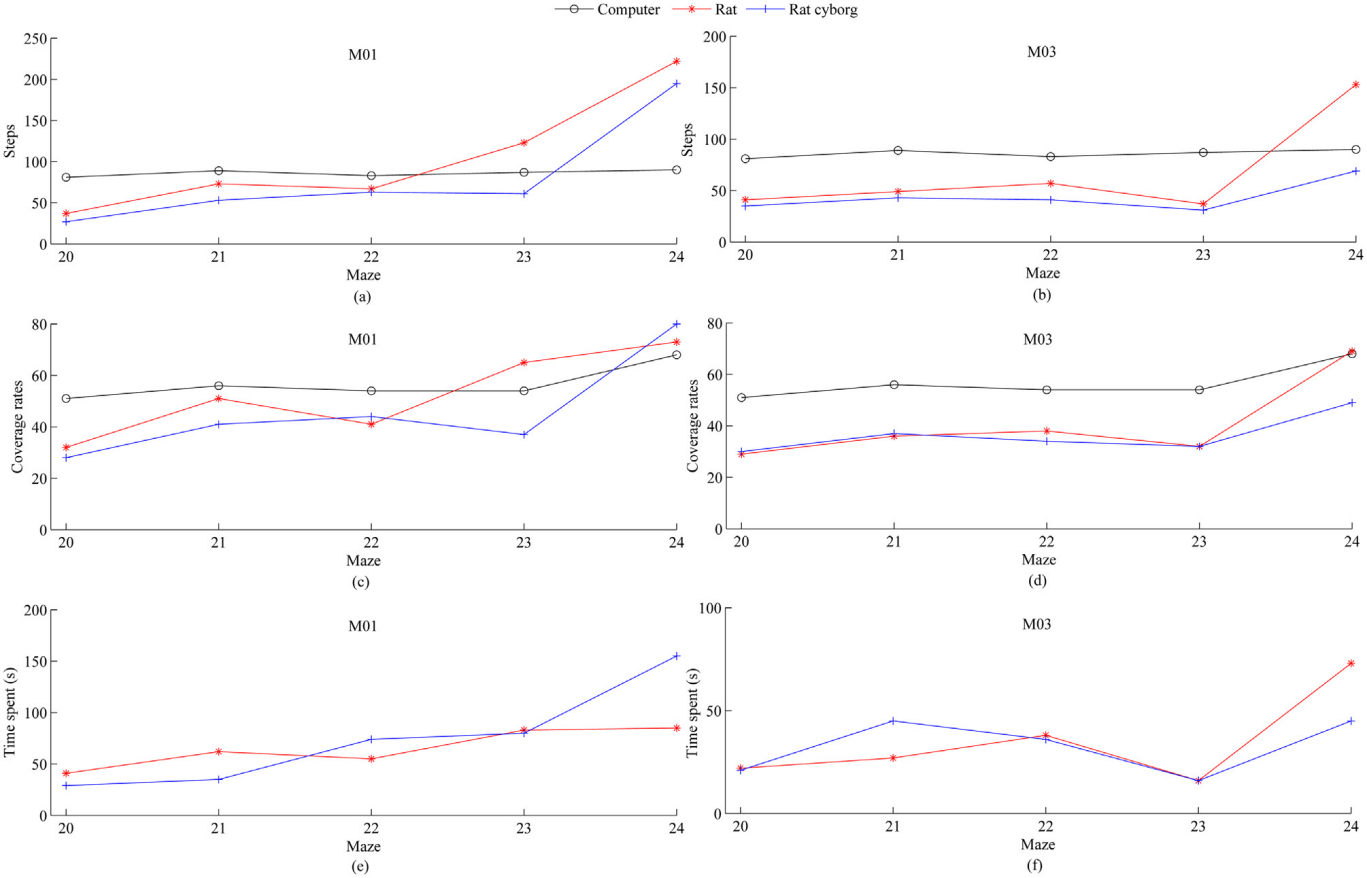
Steps, coverage rates and time spent of maze solving by computer, M01 and M03 in maze 20 to maze 24. (a) Steps of maze solving by computer and M01. (b) Steps of maze solving by computer and M03. (c) Coverage rates of maze solving by computer and M01. (d) Coverage rates of maze solving by computer and M03. (e) Time spent of maze solving by computer and M01. (f) Time spent of maze solving by computer and M03.

In supplemental experiment 2, two rats (i.e. M01 and M03) first traversed other 5 mazes (maze 20 to maze 24) one by one all by themselves. Then these 5 mazes were flipped along the diagonal axis from the entrance to the exit, and traversed by the two rats in the same order with the assistance of the computer. Note that each pair of the flipped maze and the original one (see S1 Fig) have the same complexity but different space layouts. In this way, a rat’s memory of the original maze can hardly help the counterpart rat cyborg solve the flipped maze. The experimental results are shown in Fig 10. As we can see, the steps of each rat cyborg are less than that of the corresponding rat in each maze. The coverage rates of each rat cyborg are less than that of the computer except in maze 24 of M01, and the average coverage rates of each rat cyborg (M01: 46.00±8.92, M03: 36.40±3.36) are less than that of the corresponding rat(M01: 52.40±7.52, M03: 40.80±7.22) and the computer (56.60±2.96). Besides, M03 spent less time in maze solving with the assistance of the computer except in maze 21. These results show that rat cyborgs have a better performance than rats. This is consistent with the conclusion of the previous experiments, and also demonstrates that the performance enhancement of the rat cyborgs in maze solving should not be attributed to what the rats had experienced.

Thanks to the rapid advance of brain-machine interfaces (BMIs), the connection and interaction between the organic components and computing components of the cyborg intelligent systems are becoming deeper and better [39–43]. Based on such kinds of symbiotic bio-machine systems, a new type of intelligence, which we refer to as cyborg intelligence, will play an increasingly crucial role. Cyborg intelligence is a convergence of machine and biological intelligence, which is capable of integrating the two heterogeneous intelligences at multiple levels [44, 45]. It has the potential to deliver tremendous benefits to society, such as in search and rescue, health care, and entertainment. In this work, the mobility, perceptibility and cognition capability of the rats were combined with the sensing and computing power of the machines in the rat cyborg system. The experimental results of the rat cyborg system in maze solving provide a proof-of-principle demonstration for cyborg intelligence.

## Conclusions and Future Work

In this paper, we build intelligence-augmented rat cyborgs and present a comparative study of maze solving by computer, by rats, and by rat cyborgs. Computer aids rats in dead road detection, unique road detection, loop detection, and shortest path detection. In terms of steps, coverage rates and time spent, the rat cyborgs have a better performance than the individual rats in maze solving; in terms of coverage rates, the rat cyborgs have a better performance than the individual computer in maze solving. From the systematic perspective, the rat’s capability of maze solving has been augmented by the computer.

In future work, more tasks will be introduced, and the complexity of tasks will be quantified. To avoid excessive intervention with the rats, the strength of the computer’s assistance will be graded. In addition, more practical rat cyborgs will be investigated: the web camera will be replaced by sensors mounted on rats, such as tiny camera, ultrasonic sensors, infrared sensors, electric compass, and so on, to perceive the real unknown environment in real time; and the computer-aided algorithms can be housed on a wireless backpack stimulator instead of in the computer.

## Supporting Information

S1 Fig Maps of the 24 mazes solved in our experiments. The start is in the bottom-left cell of each maze, and the target is in the top right-cell of each maze.
(PDF)

S1 File Dead road detection **(Algorithm A)**. Unique road detection **(Algorithm B)**. Loop detection **(Algorithm C)**.
(ZIP)

S2 File Maze solving by computer, rats and rat cyborgs. Computer solved maze 5 by our algorithm under the left-hand rule **(Video A)**. Computer solved maze 5 by our algorithm under the right-hand rule **(Video B)**. DH12 solved maze 5 by itself **(Video C)**. DH12 solved maze 5 with the assistance of the computer **(Video D)**.
(ZIP)

S1 Video A rat cyborg traversing complex terrain. (MP4)

S1 Table Experimental data of maze solving in the 24 mazes (maze 1 to maze 24) by our algorithm, wall follower, Pledge and Tremaux’s algorithm. (XLS)

S2 Table Experimental data of maze solving in the 14 mazes (maze 1 to maze 14) by the computer and 6 rats/rat cyborgs. (XLS)

S3 Table Experimental data of maze solving in the 10 mazes (maze 15 to maze 24) by the computer and 2 rats/rat cyborgs. (XLS)

S4 Table Stimulus parameters of the 6 rats/rat cyborgs. (XLS)
