# Supplementary material for: Intelligence-Augmented Rat Cyborgs in Maze Solving
Source: PLoS One. 2016 Feb 9;11(2):e0147754. doi: 10.1371/journal.pone.0147754 (PMC4747605; doi:10.1371/journal.pone.0147754)
Supplement: S1 File — Dead road detection (Algorithm A). Unique road detection (Algorithm B). Loop detection (Algorithm C). (ZIP) [file pone.0147754.s002.zip › S1_File/Algorithm A.pdf]

Algorithm A: Dead road detection.

```
while there is a branch not been tried do
    Let M be a 10×10 matrix of 0xff;
    Let D be a 10×10 matrix of 0;
    Let the current cell M_{cx,cy} be 0;
    select the first cell M_{x,y} in the branch;
    step ← 1;
    dead_flood_fill(x,y,step):
    M_{x,y}←step;
    step←step+1;
    if the west cell is accessible or unexplored then
        if M_{x-1,y}-M_{x,y}>1 then
            dead_flood_fill(x-1,y,step);
        end
    end
    if the north cell is accessible or unexplored then
        if M_{x,y-1}-M_{x,y}>1 then
            dead_flood_fill(x,y-1,step);
        end
    end
    if the east cell is accessible or unexplored then
        if M_{x+1,y}-M_{x,y}>1 then
            dead_flood_fill(x+1,y,step);
        end
    end
    if the south cell is accessible or unexplored then
        if M_{x,y+1}-M_{x,y}>1 then
            dead_flood_fill(x,y+1,step);
        end
    end
    return;
    if the value of target cell G is not updated (M_{9,0}=0xff) then
        for each i,j ∈{0,1,...,9} do
            if M_{i,j}≠0xff and M_{i,j}≠0 then
                D_{i,j}←1;
            end
        end
    end
end
```
