# Supplementary material for: Intelligence-Augmented Rat Cyborgs in Maze Solving
Source: PLoS One. 2016 Feb 9;11(2):e0147754. doi: 10.1371/journal.pone.0147754 (PMC4747605; doi:10.1371/journal.pone.0147754)
Supplement: S1 File — Dead road detection (Algorithm A). Unique road detection (Algorithm B). Loop detection (Algorithm C). (ZIP) [file pone.0147754.s002.zip › S1_File/Algorithm B.pdf]

Algorithm B: Unique road detection.

```
Push the target cell G into the unique cell stack T;
topcell←top element of T;
while the explore has explored a new cell do
    if the west cell of topcell is accessible and the east, north and south cell are blocked or in T then
        push the west cell into T;
    end
    else if the east cell of topcell is accessible and the west, north and south cell are blocked or in T then
        push the east cell into T;
    end
    else if the north cell of topcell is accessible and the south, west and east cell are blocked or in T then
        push the north cell into T;
    end
    else if the south cell of topcell is accessible and the north, west and east cell are blocked or in T then
        push the south cell into T;
    end
end
```
