# Supplementary material for: Intelligence-Augmented Rat Cyborgs in Maze Solving
Source: PLoS One. 2016 Feb 9;11(2):e0147754. doi: 10.1371/journal.pone.0147754 (PMC4747605; doi:10.1371/journal.pone.0147754)
Supplement: S1 File — Dead road detection (Algorithm A). Unique road detection (Algorithm B). Loop detection (Algorithm C). (ZIP) [file pone.0147754.s002.zip › S1_File/Algorithm C.pdf]

Algorithm C: Loop detection.

```
Push the start cell S into a possible exit vector V;
loopexist←flase;
while the rat cyborg has moved to another cell C do
    loopexist←flase;
    if the west cell of C is accessible and not visited then
        push the west cell into V;
    end
    if the east cell of C is accessible and not visited then
        push the east cell into V;
    end
    if the north cell of C is accessible and not visited then
        push the north cell into V;
    end
    if the south cell of C is accessible and not visited then
        push the south cell into V;
    end
    if C is in V then
        erase C from V;
    end
    if there is only one exit g in V then
        if there are more than one adjacent cells are accessible and not
         dead cell then
            loopexist←true;
        end
    end
end
```
