# Supplementary material for: Intelligence-Augmented Rat Cyborgs in Maze Solving
Source: PLoS One. 2016 Feb 9;11(2):e0147754. doi: 10.1371/journal.pone.0147754 (PMC4747605; doi:10.1371/journal.pone.0147754)
Supplement: S1 Fig — The start is in the bottom-left cell of each maze, and the target is in the top right-cell of each maze. (PDF) [file pone.0147754.s001.pdf]

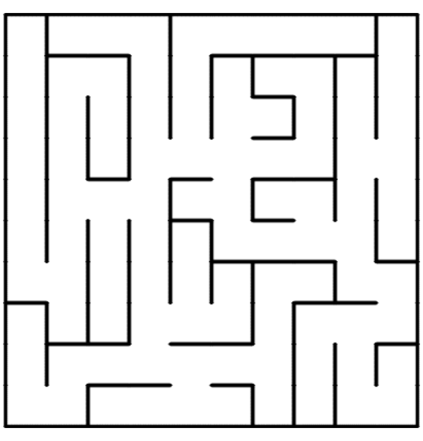
(1)

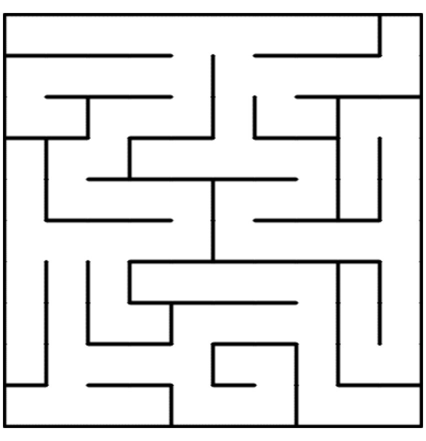
(2)

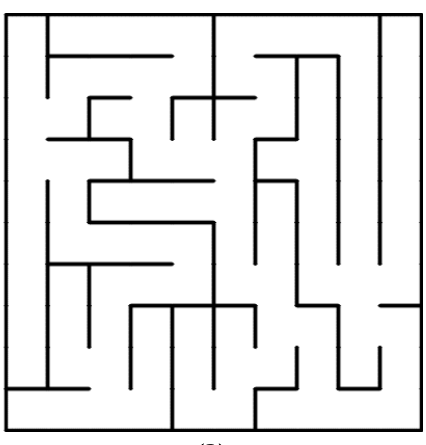
(3)

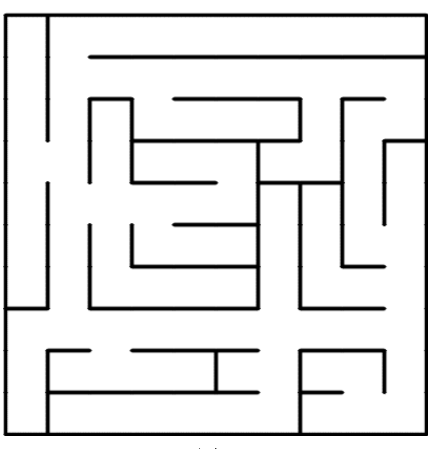
(4)

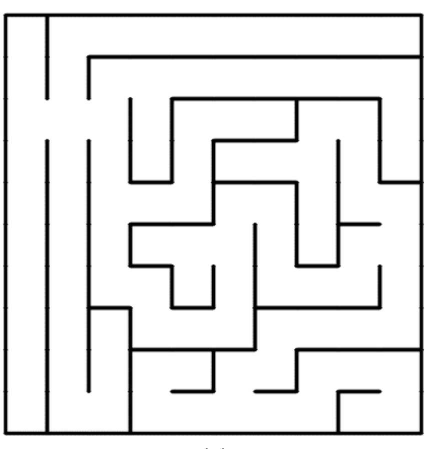
(5)

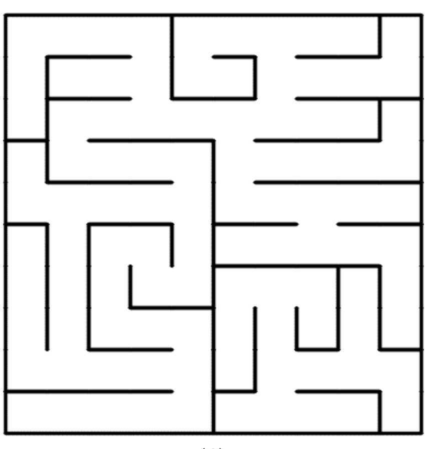
(6)

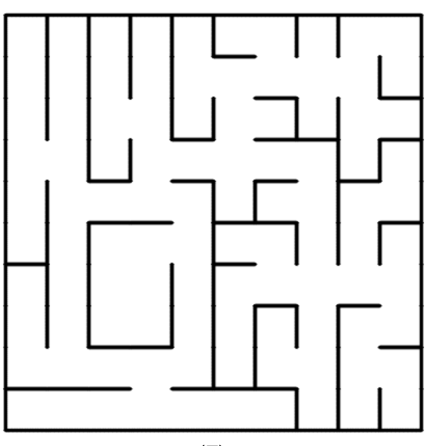
(7)

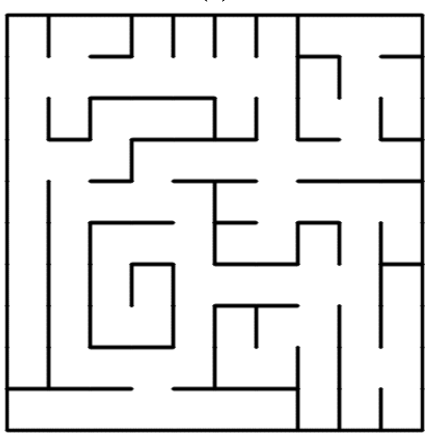
(8)

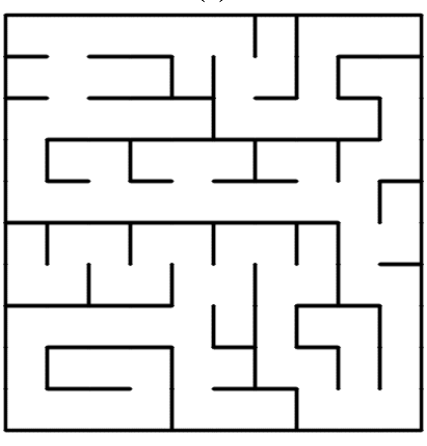
(9)

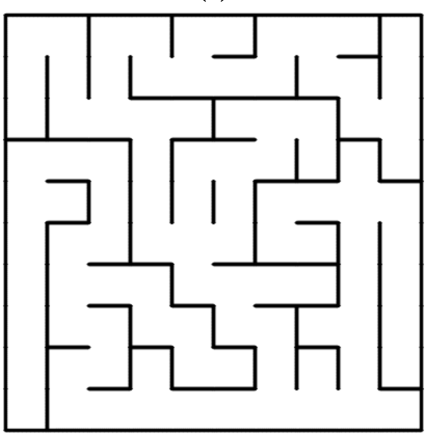
(10)

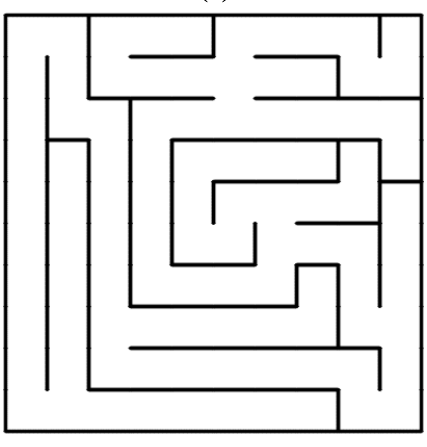
(11)

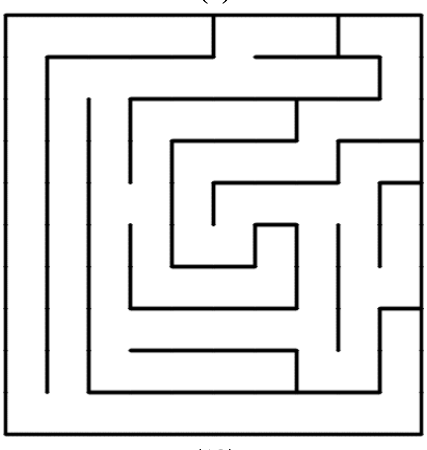
(12)

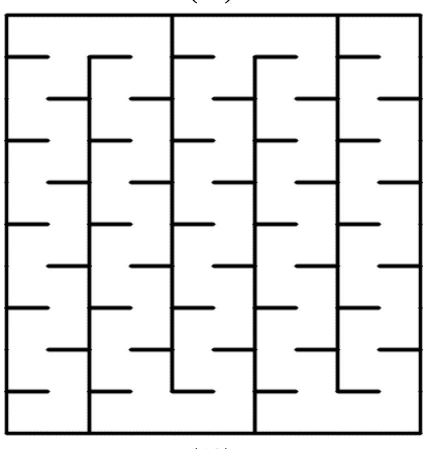
(13)

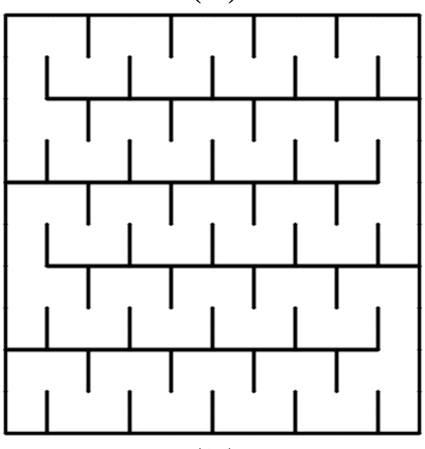
(14)

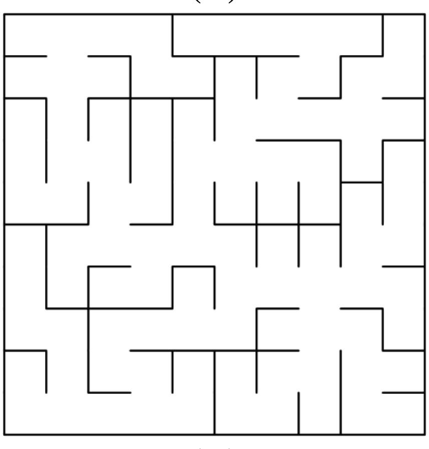
(15)

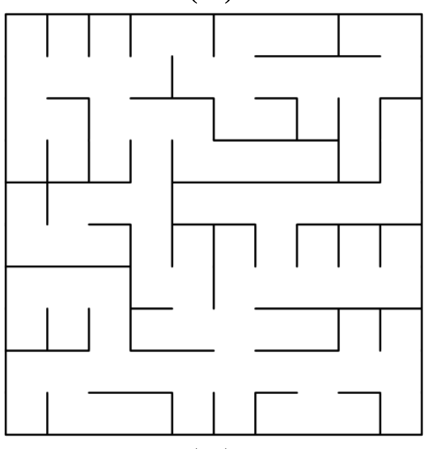
(16)

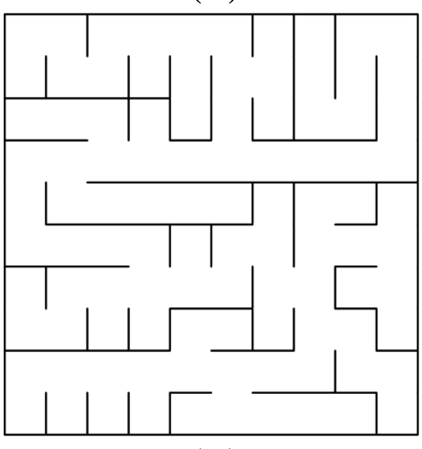
(17)

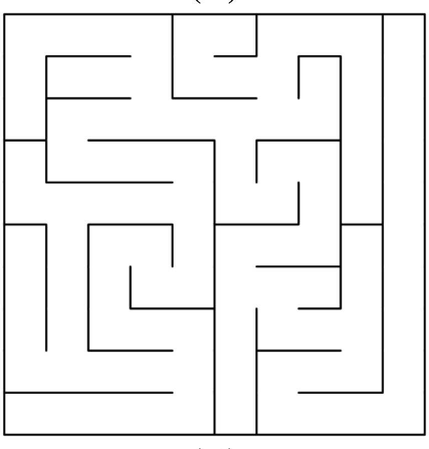
(18)

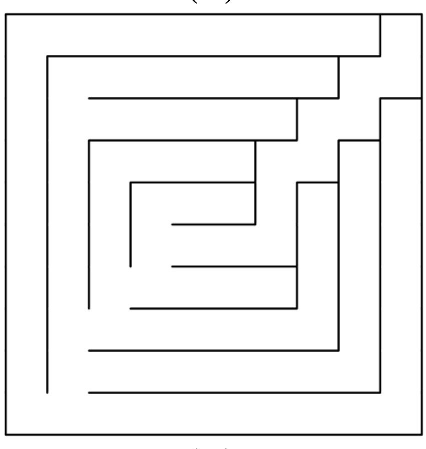
(19)

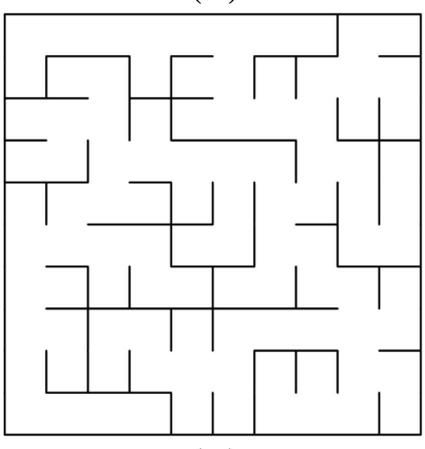
(20)

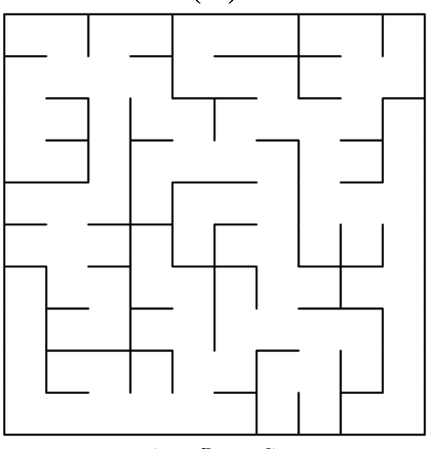
(20_flipped)

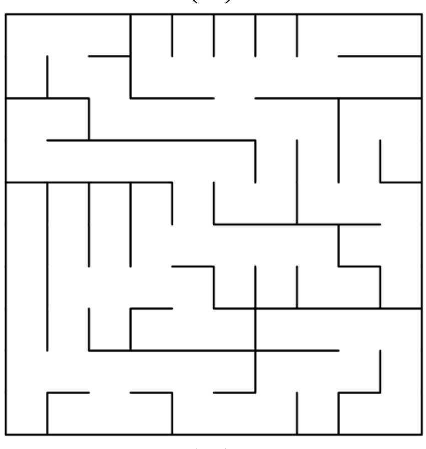
(21)

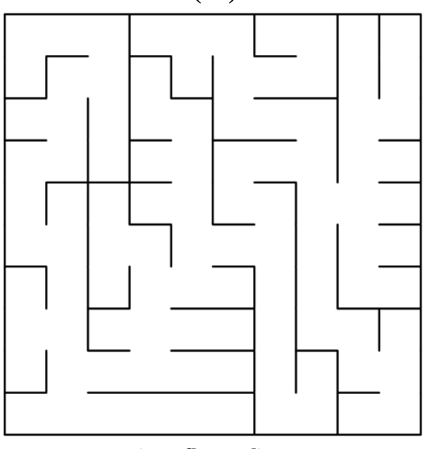
(21_flipped)

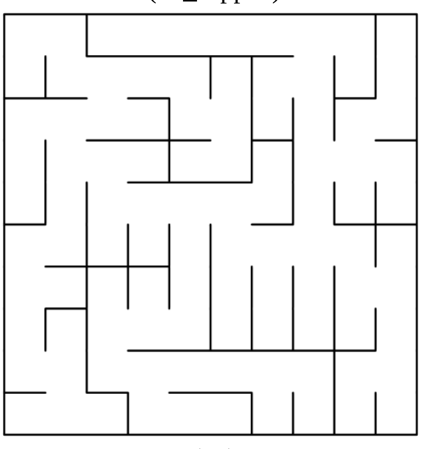
(22)

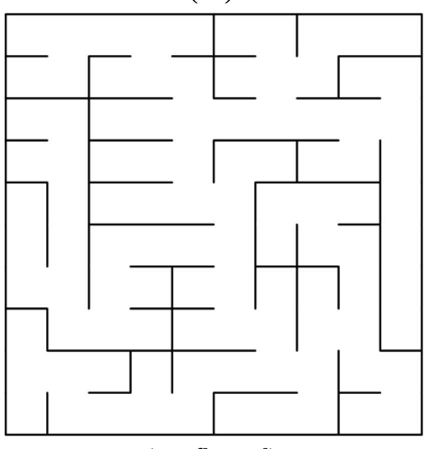
(22_flipped)

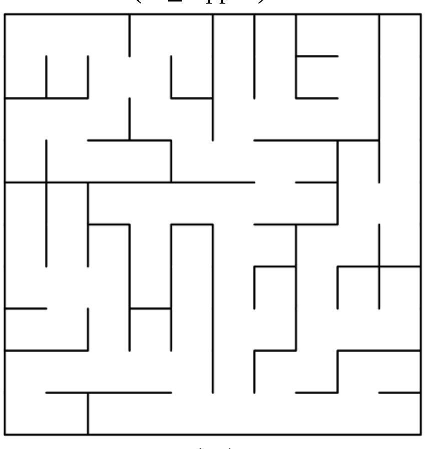
(23)

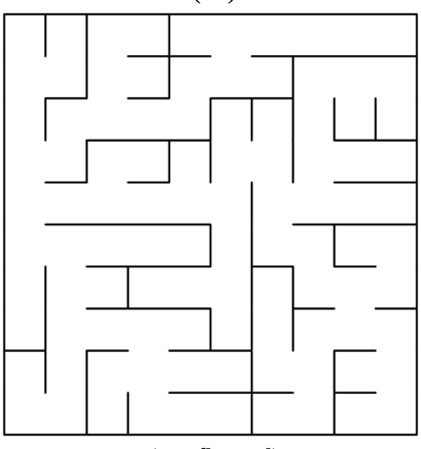
(23_flipped)

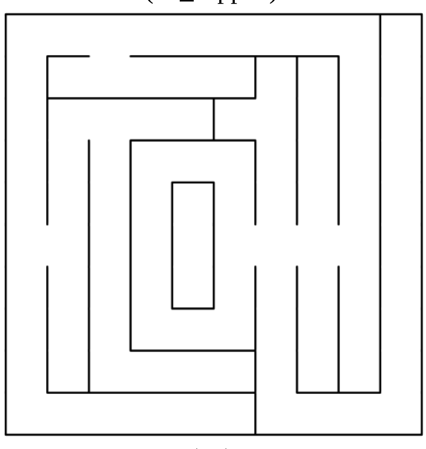
(24)

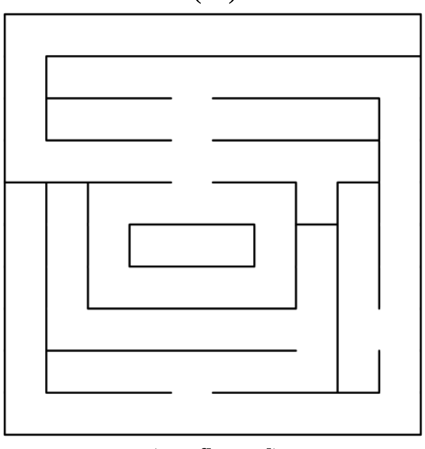
(24_flipped)
